# Effect of Stains on LDL Reduction and Liver Safety: A Systematic Review and Meta-Analysis

**DOI:** 10.1155/2018/7092414

**Published:** 2018-03-05

**Authors:** Xiao Liang, Qin He, Qinghua Zhao

**Affiliations:** Department of Hepatobiliary Surgery, The First Affiliated Hospital of Chongqing Medical University, Chongqing, China

## Abstract

**Background and Aim:**

Statin is a class of medications used to decrease low-density lipoprotein cholesterol level to prevent cardiovascular disease. However, the risk of hepatic damage caused by statin therapy is still controversial. We conducted a systematic review and meta-analysis summarizing the existing evidence of the effect of statin therapy on incidence of liver injury to clarify whether statin therapy could lead to liver function test abnormalities.

**Methods:**

We searched the Cochrane Library, PubMed, and Embase database for the relevant studies update till Jan. 2017 regarding statin therapy and liver injury. Two researchers screened the literature independently by the selection and exclusion criteria. Odds ratios (ORs) and 95% confidence intervals (CIs) were pooled using random effects models, and subgroup analyses were performed by study characteristics. This meta-analysis was performed by STATA 13.1 software.

**Results:**

Analyses were based on 74,078 individuals from 16 studies. The summary OR of statin therapy was 1.18 (95% CI: 1.01–1.39, *p* = 0.04; *I*^2^ = 0.0%) for liver injury. Subgroup analysis indicated that fluvastatin increased the risk of liver injury significantly (OR, 3.50; 95% CI: 1.07–11.53, *p* = 0.039; *I*^2^ = 0.0%) and dose over 40 mg/daily had an unfavorable effect on the liver damage (OR, 3.62; 95% CI: 1.52–8.65, *p* = 0.004; *I*^2^ = 0.0%). The sensitivity analysis indicated that the results were robust.

**Conclusion:**

Our findings confirm that statin therapy substantially increases the risk of liver injury, especially using fluvastatin over 40 mg/d.

## 1. Introduction

Statins are a group of drugs that inhibit 3-hydroxy-3-methylglutaryl coenzyme A (HMG-CoA) reductase, which is a key enzyme in the rate-limiting step in cholesterol synthesis, and are widely used since introduction in the late 1980s to reduce low-density lipoprotein (LDL) to prevent cardiovascular diseases [1]. European Guidelines on cardiovascular diseases prevention in clinical practice recommend a LDL-c goal of <100 mg/dL (2.6 mmol/L) or a ≥30% reduction in LDL-c among patients with a high risk of cardiovascular diseases [2].

However, there are some unfavorable effects of statin therapy on liver function, especially at high dose [3]. The most common clinical hepatic manifestations caused by statin are elevations in aspartate aminotransferase (AST) and alanine aminotransferase (ALT). It is reported that approximately 3% of patients showed elevations in serum aminotransferase levels exceeding three times the upper limit of normal (ULN) [4, 5]. Though the incidence rate of statin-induced liver injury reported is relatively small, it might be one of the most dangerous complications. Some rare cases even reported that statin therapy could lead to liver failure; therefore it is significantly important to figure out the statin-induced liver injury [6].

Results of the randomized control trials (RCTs) investigating the association between statin therapy and liver injury were inconsistent [7–9]. The different results may be due to the various study population, diverse types of statin, samples size, intervention duration, and dosage of the statin intervention. Previous meta-analyses summarized the effects of statin therapy on liver injury from 13 RCTs involving 49,275 participants, indicating statin therapy (pravastatin, lovastatin, and simvastatin) at low-to-moderate doses was not associated with a significant risk of liver function abnormalities [10]. There have been new RCTs published after the latest meta-analysis mentioned above; therefore, an updated meta-analysis is needed.

Our objective was to perform a comprehensive and update systematic review and meta-analysis of published RCTs, which evaluate the statin therapy (i.e., lovastatin, atorvastatin, simvastatin, pravastatin, rosuvastatin, and fluvastatin) on liver injury (AST, ALT, and ULN).

## 2. Materials and Methods

This meta-analysis was designed and reported according to the 2009 preferred reporting items for systematic reviews and meta-analysis (PRISMA) statement [11]. All analyses were based on previously published studies. Thus, no ethical approval and patients consent were required.

### 2.1. Search Strategy

Databases of Cochrane, Medline, PubMed, and Embase were searched (from inception up to Jan 2017) for studies describing the association between statin therapy and liver injury and liver function abnormalities. The search focused on MeSH terms of (atorvastatin OR simvastatin OR rosuvastatin OR fluvastatin OR pravastatin OR pitavastatin OR lovastatin OR cerivastatin OR) AND (liver injury OR liver toxicity OR hepatic injury). References and related citations of articles were manually searched for potentially eligible studies. Unpublished articles were searched from clinicaltrial.gov. All the searches were limited to human studies, and there was no language restriction in this study. Two reviewers (X L and Q W) examined all the articles, and a senior author (QH Z) was consulted when there were discrepancies about the study inclusion [12].

### 2.2. Study Selections

Trials were selected by two reviewers (X L, Q W) following the inclusion criteria: (1) human RCTs design as either parallel or crossover; (2) investigating the influence of statins on liver function; (3) trials of sample size over 150 included due to the statistical power—to obtain 80% statistical power to investigate the difference at a probability of 0.05, a sample size of 150 was needed; (4) adult participants of studies, who were over 18 years. Exclusion criteria were as follows: (1) observational studies: case-control, cross-sectional, or cohort design; (2) nonclinical studies; (3) transplant recipients; (4) infection with HIV or hepatitis B or C virus and patients with autoimmune diseases and taking immunomodulators. A senior author (QH Z) was consulted when there were discrepancies about the study inclusion.

### 2.3. Data Extraction

A standard data extraction form was used by two authors independently to collect the information, which included the first author' name, year of publication, study design, inclusion/exclusion criteria, participants' number of intervention and control group, participants' characteristics (e.g., gender, mean age), intervention details (e.g., dosage, type, frequency, and duration), placebo, the outcomes information of LFTs (e.g., ALT, AST, and ULN), case number, and compliance. A third reviewer (QH Z) was consulted when there were disagreements.

### 2.4. Quality Assessment

The quality assessment was performed by two researchers (X L, Q H) by the Cochrane Collaboration's tool [12], which included randomization procedures, allocation concealment, blinding of participants, researchers and outcome assessors, incomplete outcome data, nonelective reporting, other bias, and compliance. If all features were adequate, the quality of the studies was a low risk of bias. If one or more features were unclear, the risk of bias was unclear. If one or more features were inadequate or negative, it was at high risk of bias [12].

### 2.5. Statistical Analysis

A random effects model (using the DerSimonian-Laird method) and the generic inverse variance method were used to derive pooled estimates across studies [10]. Heterogeneity across studies was evaluated by the Cochrane *Q* statistic (significant *p* < 0.1) and the *I*^2^ statistic. An *I*^2^ statistic greater than 25% and less than 50% is considered to represent low heterogeneity. Values above 50% and less than 75% represent moderate heterogeneity and values over 75% illustrate high heterogeneity [13]. The publication bias was evaluated by Harbord method [14]. A *p* value less than 0.05 was deemed significant. Sensitivity analysis was compared between random effects model and fixed-effect model. Influence analysis method was used to perform the sensitivity test, omitting one study once till all the studies were picked out during the sensibility test. Metaregression was conducted to investigate the potential heterogeneity.

### 2.6. Subgroup Analysis

To explore the causes of inconsistency and subgroup treatment interactions, a priori subgroup analyses were designed to be conducted according to type of statin (lovastatin, atorvastatin, simvastatin, pravastatin, rosuvastatin, and fluvastatin), dose of statin (low dose, ≤20 mg/daily, moderate dose, >20 mg/daily and ≤40 mg/daily, and high dose, >40 mg/daily) [3], and duration of intervention (short-term response, less than 2 years, long-term intervention, and more than 2 years) [10]. All tests were performed using statistical software package STATA version 13.1 and two-sided *p* value < 0.05 was considered statistically significant.

## 3. Results

### 3.1. Study Identification and Studies Characteristics

**Figure 1:**
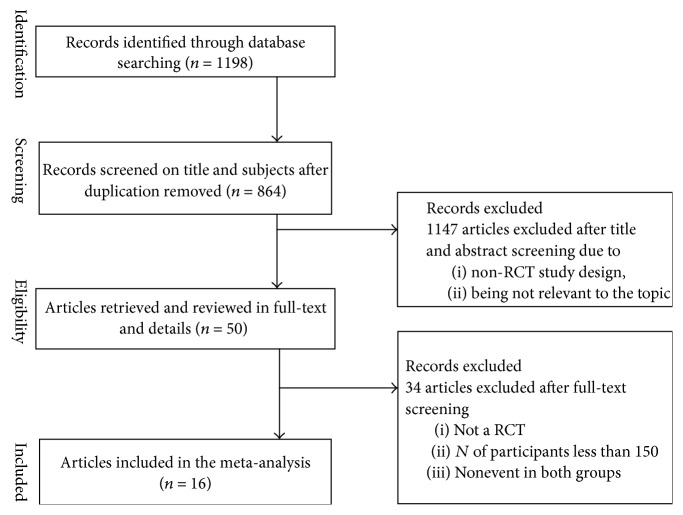
Flowchart of study selection.

We identified 74,078 participants from 16 studies in this meta-analysis [7–9, 15–27]. 1,198 published articles were found after the initial search. After the titles and abstracts screening, there were 50 articles left. Studies were excluded due to the non-RCTs study design or duplication or irrelevant references. After the full-text screening, four articles were excluded according to the fact that no LFTs abnormal events happened in both statin group and control group. Seven articles were excluded by non-RCTs study design. 23 studies were excluded because the number of participants was less than 150. The details of the studies selection process were shown in Figure 1. The statin type of this intervention included the lovastatin (*n* = 3), atorvastatin (*n* = 1), simvastatin (*n* = 5), pravastatin (5), rosuvastatin, and fluvastatin (*n* = 2). The dose of intervention in the studies ranged from 10 mg/daily to 80 ml/daily, and the duration of statin administration ranged from 3 months to 6.1 years. Description of the characteristics of the trials included is presented in Table 1. The trials reported compliance rate of 80%–100%.

### 3.2. Risk Bias of Assessment

The Cochrane Collaboration's tool for assessing the risk of bias included the adequate sequence generation, allocation concealment, blinding, incomplete outcome data, selective reporting, and another risk of bias. The accurate information of the quality of the studies was demonstrated in Table 2. Two studies were probably not allocation concealed. All of the studies have reported the exact information of the loss to follow-up and were explained in detail. Two of the studies were double-blinded properly. None of the studies was defined as a high risk of bias.

### 3.3. Meta-Analysis

**Figure 2:**
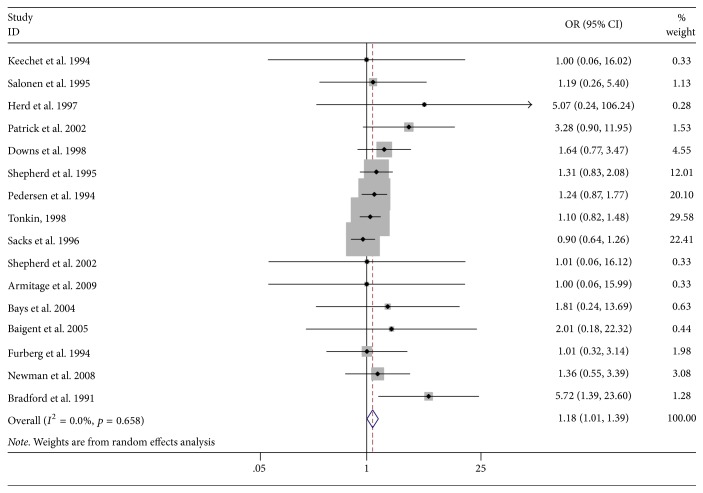
Meta-analysis of the association between statin therapy and incidence of liver injury. The area of each square is proportional to the inverse of the variance of the log relative risks. Horizontal lines represent the 95% confidence intervals (CIs). Diamonds represent pooled estimates from an inverse variance-weighted random effects model. OR = odds ratio.

The pooled odds ratio of 16 studies was 1.18 (95% CI 1.01 to 1.39, *p* = 0.04; *I*^2^ = 0.0%), indicating that statin therapy could increase the liver injury, which is shown in Figure 2.

### 3.4. Subgroup Analysis

**Figure 3:**
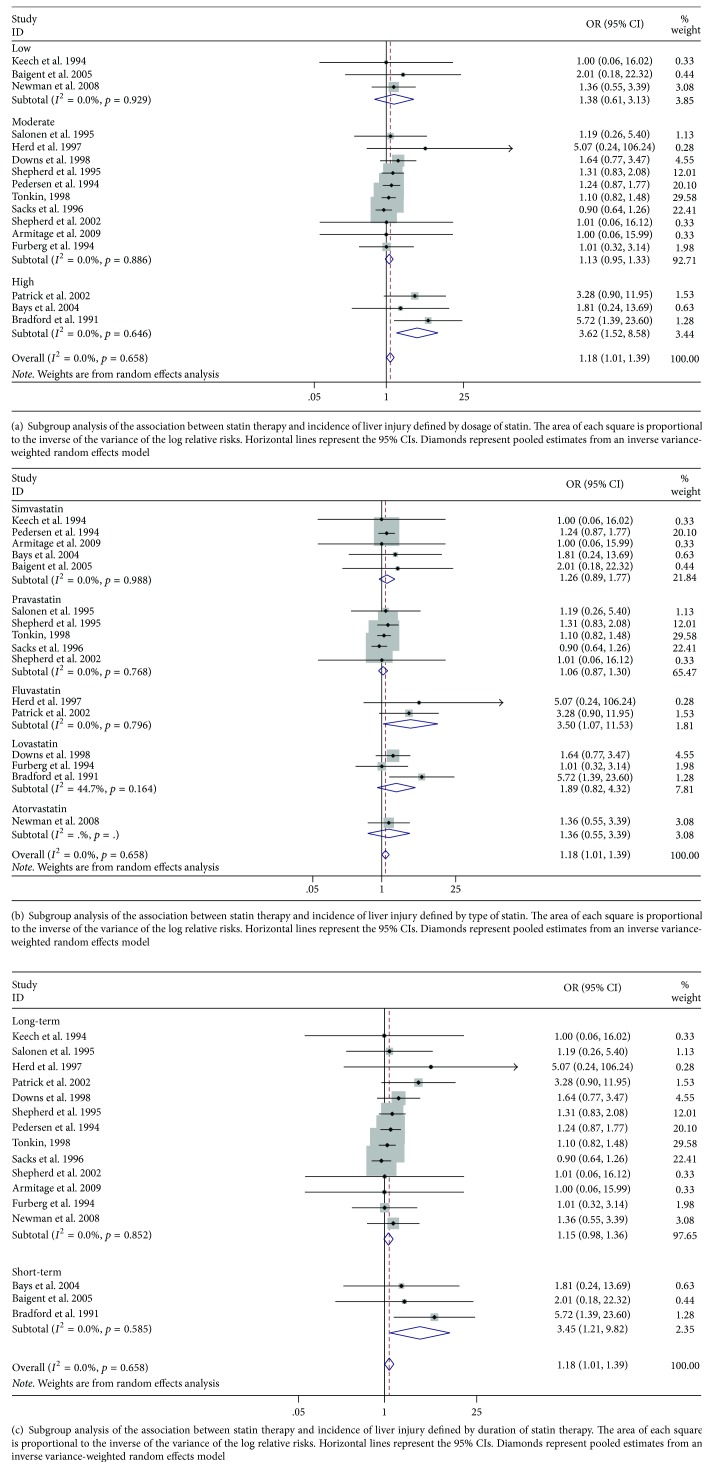


To further validate the robustness of overall analysis, subgroup analyses were carried out and are shown in Figures 3(a), 3(b), and 3(c). Fluvastatin showed an unfavorable effect on increasing the risk of liver injury (OR, 3.50; 95% CI 1.07–11.53, *p* = 0.039), while other types of statin did not show the negative effects. Daily high dose of statin therapy (over 40 mg/daily) was found to increase the risk of liver injury (OR, 3.62; 95% CI 1.52–8.58, *p* = 0.004). Moderate and low dose of the statin had no effects on liver damage. Short-term duration (less than two years) had adverse effects on liver injury rather than long-term statin therapy (OR, 3.45; 95% CI 1.21–9.82, *p* = 0.02).

### 3.5. Publication Bias

**Figure 4:**
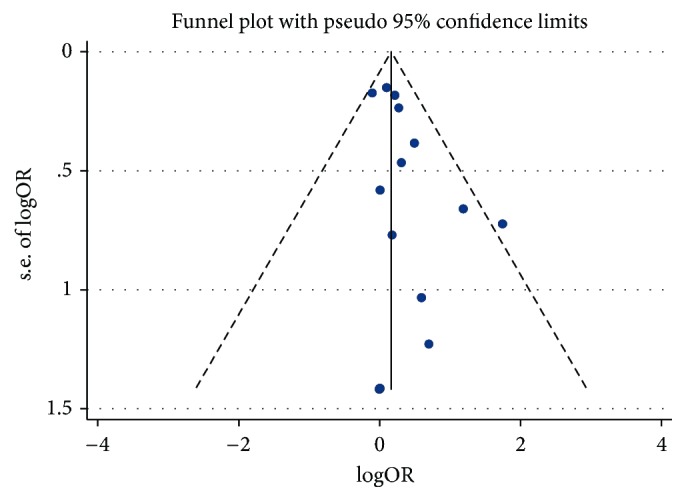
Funnel plot for statin therapy and incidence of liver injury.

Potential publication bias was not presented according to the funnel plots, which is shown in Figure 4. The Harbord tests for the studies are included in the primary analysis (*p* = 0.07), which illustrated that the small study did not contribute to the overall estimate.

### 3.6. Sensitivity Analyses and Metaregression

In sensitivity analyses the estimates of effects did not differ significantly comparing with fixed effects model (OR, 1.28; 95% CI, 1.07–1.54; *I*^2^ = 0.0%; S Figure 4) and random effects model. Influence analysis showed that one study was excluded at a time from each analysis; the results appeared to be robust to the influence of individual studies, which is shown in S Figure 1. The estimate of effect was 1.28 (95% CI, 1.07–1.54) and 1.22 (95% CI, 1.01–1.47) after excluding the most influential studies (Frank et al. and Tonkin et al.), respectively, shown in S Figures 2 and 3. There was little evidence of heterogeneity between any of these subgroups with metaregression (*p* > 0.05 by metaregression).

## 4. Discussion

The findings from this meta-analysis, based on 74,078 participants from 16 studies, illustrated that statin therapy has unfavorable effects on liver injury; the risk of liver injury was increased by 22% among patients with statin therapy. Subgroup analysis showed fluvastatin increased liver injury significantly rather than other types of statins. The results were similar to0 the previous meta-analysis conducted by Denus et al. in 2004, including 13 trials involving 49,275 patients [10]. However, there were some limitations of Denus et al.'s results. Denus et al. only included the studies with the sample size over 400 in order not to overestimate the safety of statins. Meanwhile, after calculating the statistical power of the trial as 80%, sample size of 150 is sufficient for the study. Therefore, Denus et al.'s study may overexclude some trials in the meta-analysis. Denus et al. did not perform sensitive analysis, which may not explain the heterogeneity and confirm the robustness of the results. In addition, Denus et al. only performed the subgroup analysis defined by types of statins, without addressing the issues related to the dosages and durations of statin therapy. Our comprehensive and updated meta-analysis included more trials and involved more participants. Our study did subgroup analysis according to dose of the statin and length of the intervention, which illustrated that statin over 40 mg was associated with increasing risk of liver injury. And there was no significant evidence that long-term statin intervention will increase the risk of liver injury.

Previous RCTs have indicated that daily high dose of fluvastatin was highly linked to abnormal liver enzymes [28, 29]. There was a relationship between increasing statin dose and reduction in the level of elevated triglyceride, which has been shown to be independently associated with an incidence rate of cardiovascular diseases. According to the previous studies, the incidence rate of elevation was dose dependent, with rates of 0.2% with 10 mg versus 2.3% with 80 mg [30]. Patients receiving 20 mg/daily atorvastatin showed a trend of higher transaminases than those were treated with 10 mg/daily [31]. Our study illustrates that 40 mg/daily statin therapy increases the risk of liver injury by more than 200%. Patients under statin therapy should be cautious about the dosage of intervention. Previous studies reported that there was no association between cumulative exposure and cumulative dose of statin and risk of liver injury, which illustrated that long-term use of statin might not lead to liver injury [32]. Our results also demonstrated that the negative effects were significant within 2 years rather than more than 2-year intervention. Masana et al. reported that the therapy safety and efficacy were similar between the 12th week and 48th week. The liver side effects may occur when the statins started to reduce low-density lipoprotein cholesterol within three weeks. Therefore, statin therapy should be monitored in the beginning of the intervention.

Statin is widely used to prevent cardiovascular diseases for the antithrombotic effects, cholesterol reduction, and lower risk of creatinine kinase (CK-MB) [33, 34]. Statins could reduce cardiovascular-related mortality and morbidity significantly [3]. However, one of the serious side effects of statin is liver toxicity, and it is recommended that statin therapy should be stopped if the transaminase levels are raised more than three times the upper limit of normal [35]. Although rare, statins may occasionally cause severe liver injury, some of which are with autoimmune features and some are predominantly hepatocellular [36, 37]. Statin-associated liver injury has been listed as one of the top 10 drug-associated adverse drug reactions in Taiwan [32]. According to the available guidelines when transaminase levels are raised more than three times the upper limit of normal (>3 ULN) treatment should be stopped. Potential mechanisms could explain the association between statin and liver injury.

## 5. Mechanism

The elevation of serum aminotransferase concentration is often seen in patients receiving statin therapy, especially in the first 3 months. The pathophysiologic mechanisms of hepatotoxicity are still being explored, and several potential mechanisms could explain the effects of stain on liver injury. Most people believed that the pathophysiologic mechanisms of hepatotoxicity which was caused by statins include both intracellular and extracellular mechanisms. All of these have direct effects on organelles such as the endoplasmic reticulum, mitochondria, the cytoskeleton, the nucleus, or microtubules [38]. Mitochondria dysfunction releases excessive amounts of oxidants that injure hepatic cells. Activation of some enzymes also leads to oxidative stress. They may influence cellular organelles through the activation or inhibition of signaling kinases, gene expression profiles, and transcription factors [39].

## 6. Strength and Limitation

This meta-analysis was an updated and comprehensive investigation on the effects of statin therapy and liver injury, which included more trials and larger sample size. We searched databases with no language restriction to increase the completeness of the identification of studies. We included RCTs, which avoided the influence of bias of observational studies.

Our study results should be interpreted with considerations of several potential limitations.

Firstly, there is no standard of LFTs and no specific rates of recovery of LFT abnormalities after decreasing doses from the original studies. Further studies focusing on this topic may consider individual patient data to clarify the related issue. Secondly, more than half of the trials included did not mention the methods of randomization and concealment. Two of the studies were single blinded. However, sensitivity analysis showed that the results maintained robustness. Well-designed RCTs are needed to be performed in the future. Thirdly, it has been reported that the old adults are the vulnerable group who are at high risk of cardiovascular diseases [40]. Our meta-analysis did not make a subgroup analysis according to the age. Additionally, our study did not address the issues related to the participants' disease history at baseline, such as patients with the chronic liver disease, patients with acute hepatitis, or patients with abnormal blood glucose [41, 42]. Patients suffering from active liver diseases, especially under inflammations, seem to be more vulnerable to develop impaired liver function, though there is only evidence in animal research [43]. Previous studies have reported that elevation of transaminase enzymes has frequently happened among patients with type 2 diabetes [44]. The studies in the future should analyze the basic diseases of the participants. Salmela et al. illustrated that elevated transaminase enzyme was highly related to poor diabetic management as well [44]. And the abnormal LFTs may be related to the abnormal blood glucose and insulin secretion. Therefore, statin therapy could be the single factor or could be multifactors combining with the underlying diseases, which should be clarified in the future. Finally, some of the studies included were with more than 10% of participants dropping out. However, due to the large sample size, the compliance was acceptable.

## 7. Further Implication

Although monitoring liver function tests for patients prescribed statins is recommended, the necessity of the monitoring is still questioned due to the inconsistent results.

Our meta-analysis suggests that it is essential to monitor patients receiving statin in high dose and in the beginning of intervention, especially fluvastatin, for the risk of liver injury.

## Figures and Tables

**Table 1:** The characteristic of the 16 RCTs included in our study.

Study	Year	*N*	Age	Male (%)	Statin type	Dose (mg)	Duration (year)	Compliance
Keech et al.	1994	415	63	85	Simvastatin	20	3.4	93%
Salonen et al.	1995	424	57	100	Pravastatin	40	3.2	92%
Herd et al.	1997	429	59	81	Fluvastatin	40	2.5	90%
Patrick et al.	2002	1677	60	84	Fluvastatin	80	3.9	97%
Downs et al.	1998	6605	58	85	Lovastatin	30	5.2	99%
Shepherd et al.	1995	6595	55	100	Pravastatin	40	4.9	70%
Pedersen et al.	1994	4444	59	81	Simvastatin	27	5.4	99%
Tonkin	1998	9014	62	83	Pravastatin	40	6.1	87%
Sacks et al.	1996	4159	59	86	Pravastatin	40	5.0	90%
Shepherd et al.	2002	5804	75	48	Pravastatin	40	3.2	86%
Armitage et al.	2009	20536	64	83	Simvastatin	40	5.0	85%
Bays et al.	2004	1526	55	48	Simvastatin	60	0.25	90%
Baigent et al.	2005	448	53	79	Simvastatin	20	1.0	80%
Furberg et al.	1994	919	62	52	Lovastatin	30	2.8	95%
Newman et al.	2008	2838	62	68	Atorvastatin	10	3.9	90%
Bradford et al.	1991	8245	56	59	Lovastatin	80	1.0	97%

**Table 2:** Quality assessment of the included studies.

Study	Random sequence generation	Allocation concealment	Blinding of participants personnel and outcome assessors	Incomplete outcome data	Selective outcome reporting	Other sources of bias
Keech et al. 1994	L	L	H	L	L	L
Salonen et al. 1995	L	L	L	L	L	L
Herd et al. 1997	U	U	L	L	L	U
Patrick et al. 2002	U	U	L	L	L	U
Downs et al. 1998	U	U	L	L	L	L
Shepherd et al. 1995	U	U	L	L	L	L
Pedersen et al. 1994	L	L	L	L	L	L
Tonkin 1998	U	U	L	L	L	L
Sacks et al. 1996	L	U	L	L	L	L
Shepherd et al. 2002	L	L	L	L	L	L
Armitage et al. 2009	L	L	L	L	L	L
Bays et al. 2004	U	U	H	L	L	U
Baigent et al. 2005	U	U	L	L	L	U
Furberg et al. 1994	U	U	L	L	L	L
Newman et al. 2008	U	U	L	L	L	H
Bradford et al. 1991	U	U	L	L	L	U

L: low risk of bias; H: high risk of bias; U: unclear risk of bias.

## Supplementary Materials

Supplementary Materials S. Figure 1: statin therapy and incidence of liver injury; sensitivity analysis excluding one study at a time S. Figure 2: meta-analysis of the association between statin therapy and incidence of liver injury excluding Frank's study. S. Figure 3: meta-analysis of the association between statin therapy and incidence of liver injury excluding Tonkin's study. S. Figure 4: meta-analysis of the association between statin therapy and incidence of liver injury using the fixed-effect model according to the Mantel-Haenszel (M-H) model.
