# Supplementary material for: Effect of Stains on LDL Reduction and Liver Safety: A Systematic Review and Meta-Analysis
Source: Biomed Res Int. 2018 Mar 5;2018:7092414. doi: 10.1155/2018/7092414 (PMC5859851; doi:10.1155/2018/7092414)
Supplement: Supplementary Materials — S. Figure 1: statin therapy and incidence of liver injury; sensitivity analysis excluding one study at a time S. Figure 2: meta-analysis of the association between statin therapy and incidence of liver injury excluding Frank's study. S. Figure 3: meta-analysis of the association between statin therapy and incidence of liver injury excluding Tonkin's study. S. Figure 4: meta-analysis of the association between statin therapy and incidence of liver injury using the fixed-effect model according to the Mantel-Haenszel (M-H) model. [file 7092414.f1.docx]

S. Figure 1. Statin therapy and incidence of liver injury, sensitivity analysis excluding one study at a time

S. Figure 2. Meta-analysis of the association between statin therapy and incidence of liver injury excluding Frank’s study. The area of each square is proportional to the inverse of the variance of the log relative risks. Horizontal lines represent the 95% confidence intervals (CIs). Diamonds represent pooled estimates from an inverse variance–weighted random-effects model. OR = odds ratio.

S. Figure 3. Meta-analysis of the association between statin therapy and incidence of liver injury excluding Tonkin’s study. The area of each square is proportional to the inverse of the variance of the log relative risks. Horizontal lines represent the 95% CIs. Diamonds represent pooled estimates from an inverse variance–weighted random-effects model.

S. Figure 4. Meta-analysis of the association between statin therapy and incidence of liver injury using the fixed-effect model according to the Mantel-Haenszel (M-H) model.
